# Perceptions, attitudes and experiences of hospital pharmacists working in the private sector regarding drug shortage in Lebanon: a national cross-sectional study

**DOI:** 10.1080/20523211.2025.2464786

**Published:** 2025-03-10

**Authors:** Hadi Hallal, Maha Hoteit, Souheil Hallit, Mahmoud Hallal

**Affiliations:** a Faculty of Pharmacy, Beirut Arab University, Beirut, Lebanon; b Food Science Unit, National Council for Scientific Research of Lebanon (CNRS-Lebanon), Beirut, Lebanon; c Faculty of Public Health, Lebanese University, Beirut, Lebanon; d School of Medicine and Medical Sciences, Holy Spirit University of Kaslik, Jounieh, Lebanon; e Applied Science Research Center, Applied Science Private University, Amman, Jordan; f Department of Psychology, College of Humanities, Effat University, Jeddah, Saudi Arabia; g Gastroenterology Department, Faculty of Medical Science, Lebanese University, Beirut, Lebanon; h Gastroenterology and Hepatology Department, Zahraa University Medical Center (ZHUMC), Beirut, Lebanon

**Keywords:** Lebanon, hospital pharmacists, drug shortage, experience

## Abstract

**Introduction:**

Our objective was to assess the perception, attitudes, and experiences of Lebanese hospital pharmacists in everyday practice regarding the drug shortage as well as to identify challenges and propose solutions.

**Methods:**

A cross-sectional survey was conducted between February and June 2021, enrolling 36 Lebanese hospital pharmacists.

**Results:**

The economic crisis and issues with the quality of medicines were ranked as the top two causes of drug shortages in Lebanese hospitals; 88.9% of hospital pharmacists thought that switching to lower doses and using alternatives (97.2%) can be considered viable solutions. To address the issue of drug shortages, efforts made by manufacturers were 36.1%; the availability of alternative drugs in hospitals was 63.9%. All classes of drugs were shorted in hospitals. Hospital pharmacists spent less than 5 h/day to track medicine shortages (44.5%), identifying alternatives (97.3%), purchasing alternatives (91.7%), deliberating with physicians (91.7%). Pharamcists approached ‘the parallel import' approach (75%) and contacted other hospitals (52.8%) to insure medicines alternatives.

**Discussion:**

Drug shortages in Lebanon are driven by various factors and have been increasing across governorates. Public health is the most impacted, especially for patients requiring intensive care, which consequently affects their quality of life.

## Introduction

Drug shortage is defined as a situation in which the demand for a drug exceeds its supply in pharmacies, having a significant impact on public health (Shukar et al., 2021). It is a global issue affecting low, middle, and high-income countries. The causes of shortage are multifactorial, including supply, demand and regulatory issues. Supply issues consist of manufacturing problems, unavailability of raw materials, logistic and business problems (Maddah & Maddah, 2023). Many countries have developed various strategies to address this problem, yet the issue continues to accelerate, affecting the whole world (Shukar et al., 2021). The impact of drug shortages on patient’s morbidity and mortality poses risks ranging from not being able to provide the medicine to being forced to administer a non-optimal substitute, thereby leading to potential medication errors and other complications (Faiva et al., 2021). Changing treatment when a shortage occurs introduces uncertainties as the order, preparation or dispensing procedures that are needed may also change, potentially leading to medication errors. This can breed unfamiliarity with the dosing schedules, adverse-effect profiles, and treatment efficacy for therapeutic alternatives among healthcare workers. Concerns and unfamiliarity can be enhanced if therapeutic alternatives have not been agreed in advance. To combat shortages, risks must therefore be actively differentiated based on shortage’s frequency, availability of treatment options, and provision of alternatives, without accounting for the patient’s clinical status. In Lebanon, drug shortages are nowadays one of the most encountered barriers in Lebanese hospital pharmacies. Many factors played an important role in this issue, including, but not limited to, the pharmacist’s knowledge of handling medication shortages, the economic crisis in Lebanon and the closures caused by the COVID-19 pandemic (Ammaret al., 2021a).

Pharmacists lead the logistical elements of medication shortage response as part of a multidisciplinary team from which clinical strategies emerge. There is little published guidance detailing discrete strategies to position pharmacists for an effective and accelerated response to shortages (Ammar et al., 2021b). Literature reporting the impact of pharmacist leadership in shortage response efforts has primarily focused on operational optimization, drug use policy creation, and educational efforts (Desselle et al., 2019). According to the literature, and throughout each phase of a medication shortage response, pharmacists are positioned to impact the clinical, administrative, logistical, and operational success of a health system (Ammar et al., 2021b).

There is scarce data on drug shortages in Lebanon before and amid the economic crisis. Thus, to the best of our knowledge, this is the first study of its kind showing the perceptions, attitudes and experiences of Lebanese hospital’s pharmacists amid the escalating crisis in Lebanon and identify challenges and propose solutions related to the drug shortage’s issue. The rationale for this study stems from the critical need to better understand the specific challenges Lebanese pharmacists face in the context of drug shortages. Amid the ongoing economic turmoil, the role of pharmacists in managing medication shortages has become even more pivotal yet under-researched in Lebanon. This study aims to fill this gap by examining pharmacists’ perceptions and experiences, providing insight into how they navigate this complex issue, and offering data that can help inform policies and strategies to enhance the efficiency and effectiveness of the shortage response. Furthermore, it seeks to identify key barriers to addressing shortages, such as insufficient resources, gaps in training, and coordination challenges, and propose actionable solutions tailored to the unique context of Lebanon’s healthcare system. The findings are expected to contribute to better preparedness, response strategies, and policy frameworks to mitigate the impact of drug shortages in Lebanon and similar settings.

## Methods

### Ethical considerations

Ethical approval for this study was obtained from the Institutional Review Board of the Beirut Arab University (#53; January 13, 2021). All participants were informed about the aim and scope of the study and were assured of their right to voluntary participation. Participants had the autonomy to decline participation at any point without facing any consequences. Informed consent was obtained from each participant prior to the interviews.

### Study design

This study utilized a cross-sectional survey design and was conducted between February and June 2021. In-depth interviews were carried out with Lebanese hospital pharmacists using a structured interview guide consisting of 29 questions (Appendix 1). Inclusion criteria comprised (1) licensed pharmacists working in private hospital pharmacies in Lebanon, (2) hospitals with a minimum of 30 beds in any of the Lebanese governorates, (3) pharmacists who have been employed in the hospital for at least six months, and (4) pharmacists willing to provide informed consent and participate in the study. Excluded were (1) pharmacists working in governmental hospitals, (2) pharmacists who are not licensed or practicing in Lebanese hospitals, (3) pharmacists employed for less than six months at the hospital, and (4) non-pharmacist hospital staff, including physicians, nurses, or administrative staff.

### Sample size calculation

The sample size was calculated using the Raosoft sample size calculator, assuming a population of 46 private hospital pharmacies across Lebanon with at least 30 beds each. Based on these assumptions, a sample size of at least 42 participants was deemed necessary to achieve a representative sample with a margin of error of 5% and a 95% confidence level.

### Questionnaire

The questionnaire was developed based on existing studies conducted in various countries, including Hungary (Vida et al., 2020), the United States (Chen et al., 2021), Canada (Fenna et al., 2021), and Sudan (Lucero-Prisno III et al., 2020). It contained 29 questions covering demographic information, years of experience, hospital characteristics, and questions related to the perceptions, attitudes, and practices of Lebanese pharmacists regarding drug shortages in private hospitals. Responses to the questions were categorized as ‘often,' ‘sometimes,' or ‘rarely.' For the purpose of analysis, these responses were recoded as binary variables (‘Yes' and ‘No').

To ensure the validity of the interview questions, the initial questionnaire was reviewed and validated by a panel of experts in the field of pharmacy, healthcare management, and drug shortages. These experts assessed the content for relevance, clarity, and comprehensiveness. The feedback provided by the panel was used to refine and adjust the questions to ensure they effectively captured the perceptions, attitudes, and practices of pharmacists regarding drug shortages. Additionally, the questionnaire was pilot-tested with a small group of pharmacists (*n* = 5) in Lebanon; based on their feedback, minor adjustments were made to improve the clarity and flow of the questions. The questionnaire was then finalized and translated into Arabic, with back-translation performed to preserve the intended meaning and ensure linguistic accuracy.

### Statistical analysis

The collected data was analyzed using the SPSS software (version 25.0). Descriptive statistics were used to summarize continuous variables, which were reported as means with standard deviations (SD), and categorical variables, which were reported as frequencies and percentages. The Chi-square test was applied to determine the associations between categorical variables, while Fisher’s exact test was used where applicable. *P* < .05 was predetermined to assess the statistical significance of the results.

## Results

Thirty-six hospital pharmacists were interviewed in the study: 12 pharmacists (33.3%) were from Mount Lebanon, 8 pharmacists (22.2%) from North Lebanon, 5 pharmacists (13.9%) were from south Lebanon, 5 pharmacists (13.9%) were from Beirut, 3 pharmacists (8.3%) were from Beqaa, 2 pharmacists (5.6%) were from Nabatieh and only 1 (2.8%) pharmacist was from Akkar.

### Perceptions of hospital pharmacists regarding drug shortages

**Figure 1.**
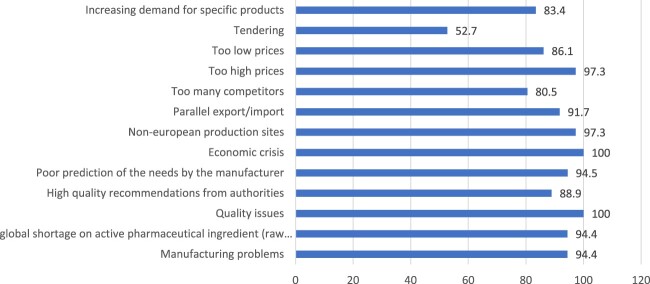
Perceptions of hospital pharmacists about drug shortages in overall hospital pharmacies.

Figure 1 shows the perceptions of hospital pharmacists regarding drug shortages in overall hospitals in the last 12 months. Our findings show that the economic crisis and quality issues ranked as top 2 causes of drug shortages in the Lebanese hospitals in this study. Supplementary Table S1 identifies the total response of hospital pharmacists and by governorate. There were no significant differences between governorates regarding the identification of causes of drug shortages in Lebanon (Table S1).

### Attitudes of hospital pharmacists concerning drug shortages

**Figure 2.**
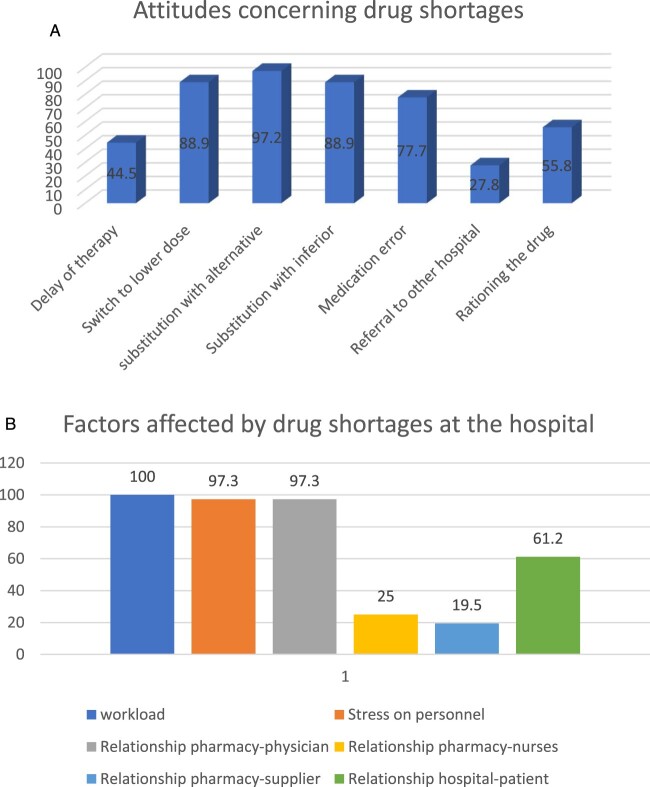
A. Attitude of hospital pharmacists to tackle drug shortages in hospital pharmacies, overall and by governorates. B. Factors affected by drug shortages at the hospital, as part of attitudes of pharmacists, overall and by governorates.

Table S2 shows the attitudes of pharmacists regarding drug shortages in their hospitals. It was shown that 80% of the pharmacists presented an impression of equality in drug shortage in all types of medicines. Around 45% of them reported delays in therapies for patients due to drug shortages. This issue is faced frequently in Akkar (100%), North Lebanon (100%) and Nabatieh (100%) (*p* = 0.03). To address this issue, 88.9% of pharmacists thought that switching to lower doses and using alternatives (97.2%) can be considered effective solutions. Hospital pharmacists in Akkar, North Lebanon and Nabatieh have always toughed about substituting drugs in case of drug shortages (100%; *p* = .039). On the other hand, most pharmacists (88.9%) thought that making substitution with inferior drugs may be considered in emergency cases. This can cause medication errors according to the interviewed pharmacists where 77.7% of them reported possible medication errors when using alternatives. Referring patients to other hospitals was rarely done in more than 70% of cases, but rationing drugs was considered by half of the participants (52.8%) (Figure 2(A)). Workload, stress on personnel and the pharmacist-physician relationship were the factors most affected by drug shortages (Figure 2(B)).

### Experiences related to the management of the drug shortages issues by the hospitals

**Figure 3.**
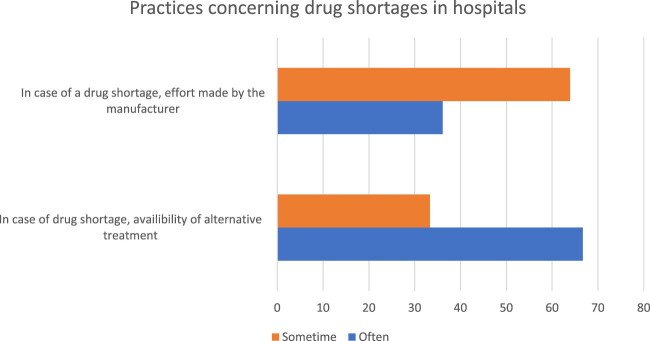
Practices concerning drug shortages in hospitals.

**Figure 4.**
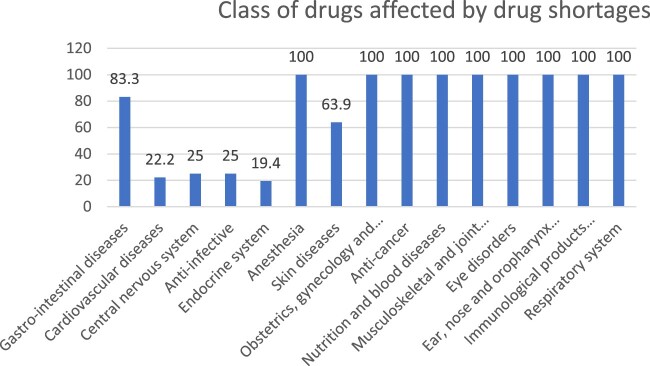
Classes of drugs affected by drug shortages according to hospital pharmacists.

In case of drug shortages, the efforts made by manufacturers were about 36.1%. On the other hand, the availability of alternative drugs in hospitals was about 63.9% as reported by hospital pharmacists (Figure 3). The class of drugs affected by drug shortages in Lebanon is presented in Figure 4. Drugs related to anesthesia, gynecology, anti-cancer, nutrition, musculoskeletal, eye disorders, ear and nose, respiratory system and immunological products were all shorted.

When participants were asked about the types of medicines in shortage, 83.3% reported that medications for gastrointestinal diseases were generic, cheap and in oral form (Table 1), 22.2% reported that the cardiovascular disease medications in shortage were generic, in oral form and ranged in price from expensive to cheap. Meanwhile, 27.8% reported that central nervous system medicines in shortage included both original and generic types, in oral and injectable forms and varied in price from expensive to cheap. Furthermore, 22.2% of the participants stated that anti-infective medicines in shortage were from both original and generic categories, with the cheapest products being in shortage. Additionally, according to participants’ answers, 5.6% of these medicines were in oral form, while 16.7% were available in both oral and dermal forms. Regarding the endocrine system, 19.4% reported that the shortage of drugs affected the originator medicines taken by oral route only, with some of these medicines being expensive and others cheap. On the other hand, 63.9% of the participants reported that expensive dermal generic medicines for skin diseases were the ones in shortage. Finally, anesthesia medications, both oral/injectable and originator/generic, were reported to be in shortage by all participants. No statistical differences were observed between governorates regarding this issue (Table 1).

**Table 1.** Practices related to drug shortages as reported by hospital pharmacists, overall and by governorates.

		Overall (*N *= 36)	Akkar (*n* = 1)	Beirut (*n *= 5)	Beqaa (*n *= 3)	Mount Lebanon (*n *= 12)	Nabatieh (*n *= 2)	North Lebanon (*n *= 8)	South Lebanon (*n *= 5)	*p*-Value
Tendering purchasing	Yes	36 (100.0%)	1 (100.0%)	5 (100.0%)	3 (100.0%)	12 (100.0%)	2 (100.0%)	8 (100.0%)	5 (100.0%)	NA
										
In case of drug shortage, is there usually an alternative treatment or product available?	Yes, often	24 (66.7%)	1 (100.0%)	4 (80.0%)	3 (100.0%)	9 (75.0%)	1 (50.0%)	2 (25.0%)	4 (80.0%)	0.139
	Yes, sometimes	12 (33.3%)	0 (0.0%)	1 (20.0%)	0 (0.0%)	3 (25.0%)	1 (50.0%)	6 (75.0%)	1 (20.0%)	
In case of a drug shortage, is an effort made by the manufacturer	Yes, always	1 (2.8%)	0 (0.0%)	1 (20.0%)	0 (0.0%)	0 (0.0%)	0 (0.0%)	0 (0.0%)	0 (0.0%)	0.451
	Yes, often	12 (33.3%)	1 (100.0%)	1 (20.0%)	1 (33.3%)	5 (41.7%)	0 (0.0%)	1 (12.5%)	3 (60.0%)	
	Yes, sometimes	21 (58.3%)	0 (0.0%)	3 (60.0%)	2 (66.7%)	5 (41.7%)	2 (100.0%)	7 (87.5%)	2 (40.0%)	
	Yes, sometimes	2 (5.6%)	0 (0.0%)	0 (0.0%)	0 (0.0%)	2 (16.7%)	0 (0.0%)	0 (0.0%)	0 (0.0%)	
*Class of drugs affected*										
Gastro-intestinal diseases	Yes	30 (83.3%)	1 (100.0%)	4 (80.0%)	2 (66.7%)	9 (75.0%)	2 (100.0%)	7 (87.5%)	5 (100.0%)	0.816
Cardiovascular diseases	Yes	8 (22.2%)	0 (0.0%)	1 (20.0%)	0 (0.0%)	4 (33.3%)	0 (0.0%)	0 (0.0%)	3 (60.0%)	0.174
Central nervous system	Yes	9 (25.0%)	0 (0.0%)	0 (0.0%)	0 (0.0%)	6 (50.0%)	0 (0.0%)	1 (12.5%)	2 (40.0%)	0.177
Anti-infective	Yes	9 (25.0%)	0 (0.0%)	1 (20.0%)	0 (0.0%)	5 (41.7%)	1 (50.0%)	2 (25.0%)	0 (0.0%)	0.48
Endocrine system	Yes	7 (19.4%)	0 (0.0%)	2 (40.0%)	0 (0.0%)	1 (8.3%)	0 (0.0%)	2 (25.0%)	2 (40.0%)	0.512
Anesthesia	Yes	36 (100.0%)	1 (100.0%)	5 (100.0%)	3 (100.0%)	12 (100.0%)	2 (100.0%)	8 (100.0%)	5 (100.0%)	NA
Skin diseases	Yes	23 (63.9%)	0 (0.0%)	4 (80.0%)	2 (66.7%)	8 (66.7%)	1 (50.0%)	4 (50.0%)	4 (80.0%)	0.706
Obstetrics, gynecology and urinary tract disorders	NR	36 (100.0%)	1 (100.0%)	5 (100.0%)	3 (100.0%)	12 (100.0%)	2 (100.0%)	8 (100.0%)	5 (100.0%)	NA
Anti-cancer	NR	36 (100.0%)	1 (100.0%)	5 (100.0%)	3 (100.0%)	12 (100.0%)	2 (100.0%)	8 (100.0%)	5 (100.0%)	NA
Nutrition and blood diseases	NR	36 (100.0%)	1 (100.0%)	5 (100.0%)	3 (100.0%)	12 (100.0%)	2 (100.0%)	8 (100.0%)	5 (100.0%)	NA
Musculoskeletal and joint diseases	NR	36 (100.0%)	1 (100.0%)	5 (100.0%)	3 (100.0%)	12 (100.0%)	2 (100.0%)	8 (100.0%)	5 (100.0%)	NA
Eye disorders	NR	36 (100.0%)	1 (100.0%)	5 (100.0%)	3 (100.0%)	12 (100.0%)	2 (100.0%)	8 (100.0%)	5 (100.0%)	NA
Ear, nose and oropharynx disorders	NR	36 (100.0%)	1 (100.0%)	5 (100.0%)	3 (100.0%)	12 (100.0%)	2 (100.0%)	8 (100.0%)	5 (100.0%)	NA
Immunological products and vaccines	NR	36 (100.0%)	1 (100.0%)	5 (100.0%)	3 (100.0%)	12 (100.0%)	2 (100.0%)	8 (100.0%)	5 (100.0%)	NA
Respiratory system	NR	36 (100.0%)	1 (100.0%)	5 (100.0%)	3 (100.0%)	12 (100.0%)	2 (100.0%)	8 (100.0%)	5 (100.0%)	NA
*Are the affected drugs mostly generic or originator?*										
Gastro-intestinal diseases	Generic	30 (83.3%)	1 (100.0%)	4 (80.0%)	2 (66.7%)	9 (75.0%)	2 (100.0%)	7 (87.5%)	5 (100.0%)	0.816
Cardiovascular diseases	Generic	8 (22.2%)	0 (0.0%)	1 (20.0%)	0 (0.0%)	4 (33.3%)	0 (0.0%)	0 (0.0%)	3 (60.0%)	0.174
Respiratory system	NR	36 (100.0%)	1 (100.0%)	5 (100.0%)	3 (100.0%)	12 (100.0%)	2 (100.0%)	8 (100.0%)	5 (100.0%)	NA
Central nervous system	Originator/generic	10 (27.8%)	0 (0.0%)	0 (0.0%)	0 (0.0%)	6 (50.0%)	1 (50.0%)	1 (12.5%)	2 (40.0%)	0.223
Anti-infective	Originator/generic	8 (22.2%)	0 (0.0%)	1 (20.0%)	0 (0.0%)	5 (41.7%)	0 (0.0%)	2 (25.0%)	0 (0.0%)	0.444
Endocrine system	Originator	7 (19.4%)	0 (0.0%)	2 (40.0%)	0 (0.0%)	1 (8.3%)	0 (0.0%)	2 (25.0%)	2 (40.0%)	0.512
Skin diseases	Generic	23 (63.9%)	0 (0.0%)	4 (80.0%)	2 (66.7%)	8 (66.7%)	1 (50.0%)	4 (50.0%)	4 (80.0%)	0.706
Anesthesia	Originator/generic	36 (100.0%)	1 (100.0%)	5 (100.0%)	3 (100.0%)	12 (100.0%)	2 (100.0%)	8 (100.0%)	5 (100.0%)	NA
Obstetrics, gynecology and urinary tract disorders	NR	36 (100.0%)	1 (100.0%)	5 (100.0%)	3 (100.0%)	12 (100.0%)	2 (100.0%)	8 (100.0%)	5 (100.0%)	NA
Anti-cancer	NR	36 (100.0%)	1 (100.0%)	5 (100.0%)	3 (100.0%)	12 (100.0%)	2 (100.0%)	8 (100.0%)	5 (100.0%)	NA
Nutrition and blood diseases	NR	36 (100.0%)	1 (100.0%)	5 (100.0%)	3 (100.0%)	12 (100.0%)	2 (100.0%)	8 (100.0%)	5 (100.0%)	NA
Musculoskeletal and joint diseases	NR	36 (100.0%)	1 (100.0%)	5 (100.0%)	3 (100.0%)	12 (100.0%)	2 (100.0%)	8 (100.0%)	5 (100.0%)	NA
Eye disorders	NR	36 (100.0%)	1 (100.0%)	5 (100.0%)	3 (100.0%)	12 (100.0%)	2 (100.0%)	8 (100.0%)	5 (100.0%)	NA
Ear, nose and oropharynx disorders	NR	36 (100.0%)	1 (100.0%)	5 (100.0%)	3 (100.0%)	12 (100.0%)	2 (100.0%)	8 (100.0%)	5 (100.0%)	NA
Immunological products and vaccines	NR	36 (100.0%)	1 (100.0%)	5 (100.0%)	3 (100.0%)	12 (100.0%)	2 (100.0%)	8 (100.0%)	5 (100.0%)	NA
*Are the affected drugs mostly cheap or expensive?*										
Gastro-intestinal diseases	Cheap	30 (83.3%)	1 (100.0%)	4 (80.0%)	2 (66.7%)	9 (75.0%)	2 (100.0%)	7 (87.5%)	5 (100.0%)	0.816
Cardiovascular diseases	Cheap/expensive	8 (22.2%)	0 (0.0%)	1 (20.0%)	0 (0.0%)	4 (33.3%)	0 (0.0%)	0 (0.0%)	3 (60.0%)	0.174
Central nervous system	Cheap/expensive	10 (27.8%)	0 (0.0%)	0 (0.0%)	0 (0.0%)	6 (50.0%)	1 (50.0%)	1 (12.5%)	2 (40.0%)	0.223
Anti-infective	Cheap	8 (22.2%)	0 (0.0%)	1 (20.0%)	0 (0.0%)	5 (41.7%)	0 (0.0%)	2 (25.0%)	0 (0.0%)	0.444
Skin diseases	Expensive	24 (66.7%)	0 (0.0%)	4 (80.0%)	2 (66.7%)	8 (66.7%)	1 (50.0%)	5 (62.5%)	4 (80.0%)	0.795
Endocrine system	Cheap/expensive	7 (19.4%)	0 (0.0%)	2 (40.0%)	0 (0.0%)	1 (8.3%)	0 (0.0%)	2 (25.0%)	2 (40.0%)	0.512
Anesthesia	Cheap	36 (100.0%)	1 (100.0%)	5 (100.0%)	3 (100.0%)	12 (100.0%)	2 (100.0%)	8 (100.0%)	5 (100.0%)	NA
Respiratory system	NR	36 (100.0%)	1 (100.0%)	5 (100.0%)	3 (100.0%)	12 (100.0%)	2 (100.0%)	8 (100.0%)	5 (100.0%)	NA
Obstetrics, gynecology and urinary tract disorders	NR	36 (100.0%)	1 (100.0%)	5 (100.0%)	3 (100.0%)	12 (100.0%)	2 (100.0%)	8 (100.0%)	5 (100.0%)	NA
Anti-cancer	NR	36 (100.0%)	1 (100.0%)	5 (100.0%)	3 (100.0%)	12 (100.0%)	2 (100.0%)	8 (100.0%)	5 (100.0%)	NA
Nutrition and blood diseases	NR	36 (100.0%)	1 (100.0%)	5 (100.0%)	3 (100.0%)	12 (100.0%)	2 (100.0%)	8 (100.0%)	5 (100.0%)	NA
Musculoskeletal and joint diseases	NR	36 (100.0%)	1 (100.0%)	5 (100.0%)	3 (100.0%)	12 (100.0%)	2 (100.0%)	8 (100.0%)	5 (100.0%)	NA
Eye disorders	NR	36 (100.0%)	1 (100.0%)	5 (100.0%)	3 (100.0%)	12 (100.0%)	2 (100.0%)	8 (100.0%)	5 (100.0%)	NA
Ear, nose and oropharynx disorders	NR	36 (100.0%)	1 (100.0%)	5 (100.0%)	3 (100.0%)	12 (100.0%)	2 (100.0%)	8 (100.0%)	5 (100.0%)	NA
Immunological products and vaccines	NR	36 (100.0%)	1 (100.0%)	5 (100.0%)	3 (100.0%)	12 (100.0%)	2 (100.0%)	8 (100.0%)	5 (100.0%)	NA
*What is the most affected form?*										
Gastro-intestinal diseases	Oral	30 (83.3%)	1 (100.0%)	4 (80.0%)	2 (66.7%)	9 (75.0%)	2 (100.0%)	7 (87.5%)	5 (100.0%)	0.816
Cardiovascular diseases	Oral	8 (22.2%)	0 (0.0%)	1 (20.0%)	0 (0.0%)	4 (33.3%)	0 (0.0%)	0 (0.0%)	3 (60.0%)	0.174
Anesthesia	Oral/injectable	36 (100.0%)	1 (100.0%)	5 (100.0%)	3 (100.0%)	12 (100.0%)	2 (100.0%)	8 (100.0%)	5 (100.0%)	NA
Central nervous system	Oral/injectable	10 (27.8%)	0 (0.0%)	0 (0.0%)	0 (0.0%)	6 (50.0%)	1 (50.0%)	1 (12.5%)	2 (40.0%)	0.223
Anti-infective	Oral	2 (5.6%)	0 (0.0%)	0 (0.0%)	0 (0.0%)	2 (16.7%)	0 (0.0%)	0 (0.0%)	0 (0.0%)	0.786
	Oral/dermal	6 (16.7%)	0 (0.0%)	1 (20.0%)	0 (0.0%)	3 (25.0%)	0 (0.0%)	2 (25.0%)	0 (0.0%)	
Endocrine system	Oral	7 (19.4%)	0 (0.0%)	2 (40.0%)	0 (0.0%)	1 (8.3%)	0 (0.0%)	2 (25.0%)	2 (40.0%)	0.512
Skin diseases	Dermal	23 (63.9%)	0 (0.0%)	4 (80.0%)	2 (66.7%)	8 (66.7%)	1 (50.0%)	4 (50.0%)	4 (80.0%)	0.706
Respiratory system	NR	36 (100.0%)	1 (100.0%)	5 (100.0%)	3 (100.0%)	12 (100.0%)	2 (100.0%)	8 (100.0%)	5 (100.0%)	NA
Obstetrics, gynecology and urinary tract disorders	NR	36 (100.0%)	1 (100.0%)	5 (100.0%)	3 (100.0%)	12 (100.0%)	2 (100.0%)	8 (100.0%)	5 (100.0%)	NA
Anti-cancer	NR	36 (100.0%)	1 (100.0%)	5 (100.0%)	3 (100.0%)	12 (100.0%)	2 (100.0%)	8 (100.0%)	5 (100.0%)	NA
Nutrition and blood diseases	NR	36 (100.0%)	1 (100.0%)	5 (100.0%)	3 (100.0%)	12 (100.0%)	2 (100.0%)	8 (100.0%)	5 (100.0%)	NA
Musculoskeletal and joint diseases	NR	36 (100.0%)	1 (100.0%)	5 (100.0%)	3 (100.0%)	12 (100.0%)	2 (100.0%)	8 (100.0%)	5 (100.0%)	NA
Eye disorders	NR	36 (100.0%)	1 (100.0%)	5 (100.0%)	3 (100.0%)	12 (100.0%)	2 (100.0%)	8 (100.0%)	5 (100.0%)	NA
Ear, nose and oropharynx disorders	NR	36 (100.0%)	1 (100.0%)	5 (100.0%)	3 (100.0%)	12 (100.0%)	2 (100.0%)	8 (100.0%)	5 (100.0%)	NA
Immunological products and vaccines	NR	36 (100.0%)	1 (100.0%)	5 (100.0%)	3 (100.0%)	12 (100.0%)	2 (100.0%)	8 (100.0%)	5 (100.0%)	NA

**Figure 5.**
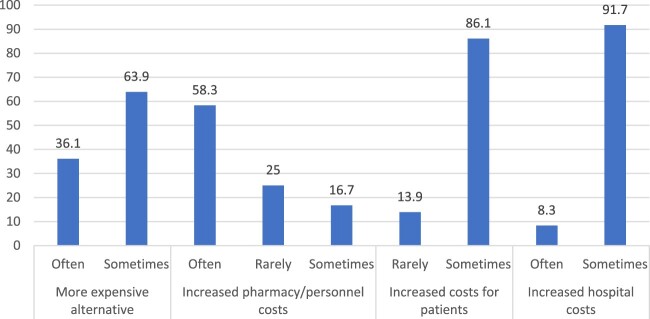
Financial consequences of drug shortages on hospitals.

Drug shortages also had an impact on the financial status of hospitals. According to Figure 5, in most cases, hospitals switched to more expensive alternative medicines to address the shortage, which increased the overall pharmaceutical costs at the hospital by 60%. This also resulted in an 86.1% increase in costs at the patient level and a 91.7% increase at the hospital pharmacy level (Table 1).

### Experiences of hospital pharmacists with regards to drug shortages in the hospitals

It appears that the majority of pharmacists in hospitals spend a timing of less than 5 h per day to track medicine shortages (44.5%), identify alternatives (97.3%), purchase alternatives (91.7%), deliberate with physicians (91.7), educate nursing staff (63.9%), develop policies (50%) and change stocks in the pharmacy and departments (53.8%).

**Figure 6.**
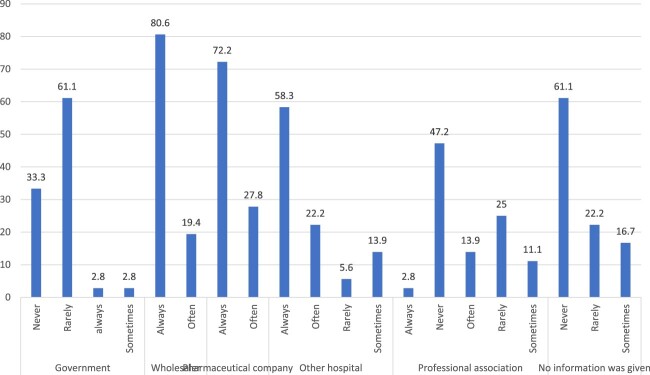
Sources of information related to drug shortages in Lebanese hospitals.

The source of information related to drug shortages is shown in Figure 6. Based on participants’ answers, 61% reported a lack of information regarding drug shortages. Moreover, 94.4% reported no announcements from the government, and around half indicated a lack of information dissemination by professional associations. The main sources of information about drug shortages were wholesalers (80.6%), pharmaceutical companies (72.2%) and other hospitals (58.3%).

**Figure 7.**
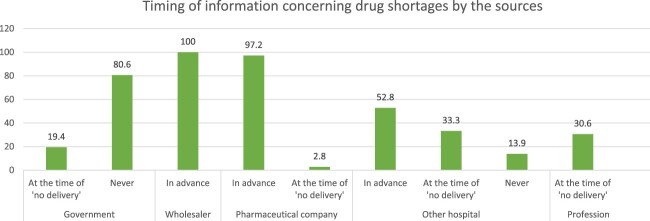
Timing of information by the pharmaceutical parties in concern with drug shortages.

**Figure 8.**
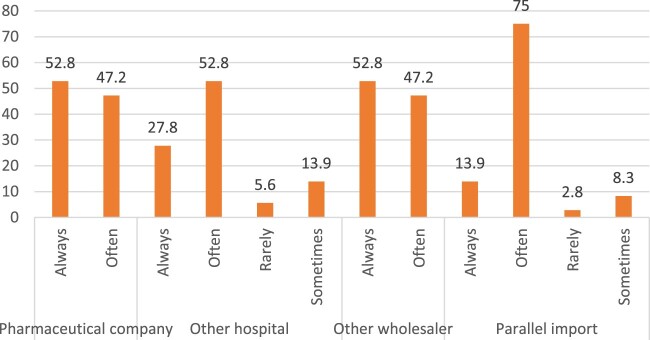
Channels accessed by hospital pharmacists to tackle drug shortages crisis.

According to Figure 7, 19.4% and 30.6% of participants reported being informed about medicine shortages by the government and professional associations, respectively, at the time of the shortages. On the other hand, wholesalers and pharamceutical companies ranked first in reporting this issue in advance. Half of the participants reported learning about drug shortages from other hospitals in advance. Figure 8 shows the channels hospital pharmacists used during a drug shortage episode. Pharamcetutical companies and other wholesalers were the most frequently approached sources (52.8% for both). Hospital pharmacies often resorted to ‘the parallel import' approach (75%) and contacting other hospitals (52.8%).

## Discussion

To the best of our knowledge, this is the first study of its type in the country. The economic crisis imposed itself on the medicines’ quality, which both caused drug shortages for around 80% of medicines in the Lebanese hospitals.

### Classes of drugs in shortages in Lebanon in comparison with international data

All classes of drugs, from all types (generic/originator, oral/injectable/dermal) and at all prices, related to anesthesia, gynecology, anti-cancer, nutrition, musculoskeletal, eye disorders, ear and nose, respiratory system and immunological products were shorted in Lebanese hospitals at the time of this study. This scenario has been frequently observed in many countries worldwide. Nearly all types of drugs have been reported in shortage, including antibiotics (A07AA/D01AA/G01AA/J02AA/S01AA), antiretroviral drugs (J05AR), anti-protozoal (P01), antineoplastic agents (L01), cardiovascular medicines (C), analgesics (N02), etc. Different countries or areas encounter different drugs in shortage depending on their health conditions and needs (Khattar et al., 2022; Mazer-Amirshahi et al., 2020; Mazer-Amirshahi et al., 2014; Rinaldi et al., 2017). However, essential (World Health Organization 2016) and emergency medicines (Alsirafy & Farag, 2016; Benge & Burka, 2019; Dill & Ahn, 2014; Fox & McLaughlin, 2018; Mazer-Amirshahi et al., 2014) are more likely to experience shortages compared to other types of medicines. In high-income countries, research studies identified different classes of drugs in short supply. However, there are fewer studies in low – and middle-income countries, which provide a complete picture of drug shortages. Only a few studies focus on the affordability, availability, and shortage of essential medicines in these regions (Phuong et al., 2019). Almost all classes of medicines were in shortage in high-income countries. Antimicrobial agents are the most affected class by drug shortage (Mazer-Amirshahi et al., 2017), along with oncology drugs (both chemotherapeutic (D06B/ D06C/D06BX) and non-chemotherapeutic drugs) (Costelloe et al., 2015; Woodcock & Wosinska, 2013).

In low-income countries, the literature often cites shortages of medicines used for tuberculosis, malaria, and HIV due to the increased incidence of these diseases and the unavailability of essential drugs rather than traditional drug shortages or stock-outs. During the COVID-19 pandemic, shortages of active pharmaceutical ingredients (APIs), excipients, and drugs occurred worldwide. As a result, countries like India, China, and the United States, which are the major producers of APIs, stopped supplying certain APIs to other countries, which led to a global shortage of many drugs. Moreover, many other challenges, including shortages of packaging materials, disrupted transportation, delayed shipping and customer clearance, restricted import-export of APIs and drugs worldwide (Ayati et al., 2020; Badreldin & Atallah, 2021). Moreover, during the COVID-19 pandemic, shortages of API were also found in the United States (Coustasse et al., 2020). When a sole supplier provides the API and excipients for any product, any problem with that supplier may lead to a medicine shortage (Shukar et al., 2021). Therefore, having at least three suppliers for materials is usually considered desirable to prevent such shortages (World Health Organization 2016). The unavailability of raw materials has been identified as an important reason for drug shortages in Saudi Arabia (Alsheikh et al., 2016), Canada (Videau et al., 2019), and Pakistan (Atif et al., 2019).

### Mitigation measures conducted by hospital pharmacists

In the current study, 88.9% of hospital pharmacists reported that mitigation measures for drug shortages included switching to lower doses and using alternatives (97.2%). Besides, most pharmacists (88.9%) thought that substituting with inferior drug could be considered in emergency cases. Referring patients to other hospitals was rarely done in more than 70% of cases, while rationing drugs was considered by half of the participants (52.8%). To address the drug shortage issue, efforts made by manufacturers were about 36.1%, and the availability of alternative drugs in hospitals was about 63.9% as reported by hospital pharmacists. Despite the serious challenge posed by drug shortages, hospital pharmacists spent less than 5 h per day to track medicine shortages (44.5%), identify alternatives (97.3%), purchase alternatives (91.7%), deliberate with physicians (91.7), educate nursing staff (63.9%), develop policies (50%) and change stocks in the pharmacy and departments (53.8%). In addition, the main channels approached by the hospital pharmacists were pharmaceutical companies and other wholesalers (52.8% for both). Hospital pharmacies often used ‘the parallel import' approach (75%) and contacted other hospitals (52.8%) to ensure the availability of alternative medicines.

Drug shortages are well-studied in the United States (South and North America), European Union, Oceania countries (Fiji, Australia) (Acosta et al., 2019; Shukar et al., 2021). In contrast, few related studies were found in Asia (Saudi Arabia, Pakistan, China, Iran, Iraq and Jordan) and Africa (Kenya, South Africa, Egypt, and Uganda) (Acosta et al., 2019; Shukar et al., 2021). Although extensive work has been done to implement policies for drug shortages mitigation in the United States and European countries, there is an extensive research gap still in the rest of the world (Acosta et al., 2019). Different strategies are proposed in most of the high-income and some middle-income countries to cope with drug shortages. Drug shortages international and national organizations, including the World Health Organization (WHO), the International Pharmaceutical Federation (FIP), American Society of Health-System Pharmacist (ASHP), and the European Association of Hospital Pharmacists (EAHP), are extensively involved in taking initiatives and providing information and guidelines to mitigate the drug shortage situation as shown in Figure 3 (Food and Drug Administration, 2013). Simultaneously, many approaches have been proposed by the United States, European countries, Canada, Australia, China, and others. However, this problem persists and remains overlooked in low- and middle-income countries. Therefore, there is a need for comprehensive, universally applicable and updated strategies to address this issue internationally. In hospitals, the healthcare team uses the following strategies for the management of drug shortages: (1) informing prescribers and recommending alternative agents, (2) contacting other suppliers for the missing medicine, (3) investigating supply restoration and planning, (4) substituting the prescribed medication and (5) updating the formulary. However, in community pharmacies, community pharmacists and working staff adopt the following strategies: (1) managing current shortages, (2) contacting the authorized supplier, (3) contacting other pharmacies, and (4) suggesting an alternative treatment to the patient (Panic et al., 2020; Tan et al., 2016).

### Drug shortages and medication errors

Drug shortages can cause medication errors according to the interviewed hospital’s pharmacists where 77.7% reported possible medication errors when using alternatives. Clinical outcomes of drug shortage have been reported in the majority of studies in developed countries, including alterations in treatment, inferior treatment, prescription inaccuracies, dispensing errors, administration errors, delayed or denied treatment, prolonged hospitalization, adverse drug interactions, and even death as reported in the United States, Saudi Arabia, Europe, Australia, Canada, United Kingdom (Alsheikh et al., 2016; Becker et al., 2013; Bero et al., 2017; Dave et al., 2018; Dill & Ahn, 2014; Fox et al., 2014; McLaughlin et al., 2013, 2017; Phuong et al., 2019; Rinaldi et al., 2017). A survey conducted in North Carolina, South Carolina, Georgia, and Florida reported that drug shortage caused a significant percentage of medication errors in patients, resulting in compromised health outcomes and increased patient burden. This created an unsafe situation for both staff and patients. For example, Alfuzosin was used as a replacement Tamsulosin, which was in short supply; however, Alfuzosin increases QT interval, which poses additional risks to patients (Hsia et al., 2015; Shaban et al., 2018). The shortage of antimicrobial drugs is critical as their shortages lead to delayed treatment, chronic infection, and other deadly outcomes (Fox et al., 2014). In addition, some drugs in the grey market become substandard over time when stored in non-optimal conditions, which can lead to compromised health outcomes (Fox et al., 2014; Zwaida et al., 2019). The available online medicines may have quality problems. Many studies found that online purchasing is common in high-income countries like Malta, the United Kingdom, and the Netherlands, where it carries an increased risk of counterfeit medicines and higher drug prices compared to local purchasing (Fittler et al., 2018; Koenraadt & van de Ven, 2018). Drug shortages lead to inappropriate alternatives in prescription, compromised health, prolonged hospital stay, readmission, morbidity, and mortality in developing countries (Uganda, Fiji, Zambia, Nigeria, Egypt) (Bero et al., 2017; Malik et al., 2013). Studies have proven that interrupted treatments due to drug shortages for antiretroviral therapy (ART) led to substandard outcomes, accumulations of drug resistance mutations, and treatment failure (Meloni et al., 2017). For some critical medicines, their shortage can lead to the cancellation of surgeries. For example, the shortage of protamine sulfate may result in the cancellation of heart surgery. Such cancellation can worsen the patient’s condition, prolong their hospital stay, and increase the risk of hospital-acquired infections (Burki, 2017; Khan, 2019). More seriously, the shortage of certain medicines may lead to higher mortality rates. For example, the shortage of chemotherapeutic drugs has been linked to a higher mortality rate, while shortages of essential drugs, including antibiotics, phytonadione, electrolyte solutions, analgesics, and opioids, have also contributed to increased patient harm (Mazer-Amirshahi et al., 2014). Drug shortages also increased the online purchasing of counterfeit products, particularly in middle-income countries (Huang & Xu, 2017; Yin et al., 2016).

### Economic impact of drug shortages

**Figure 9.**
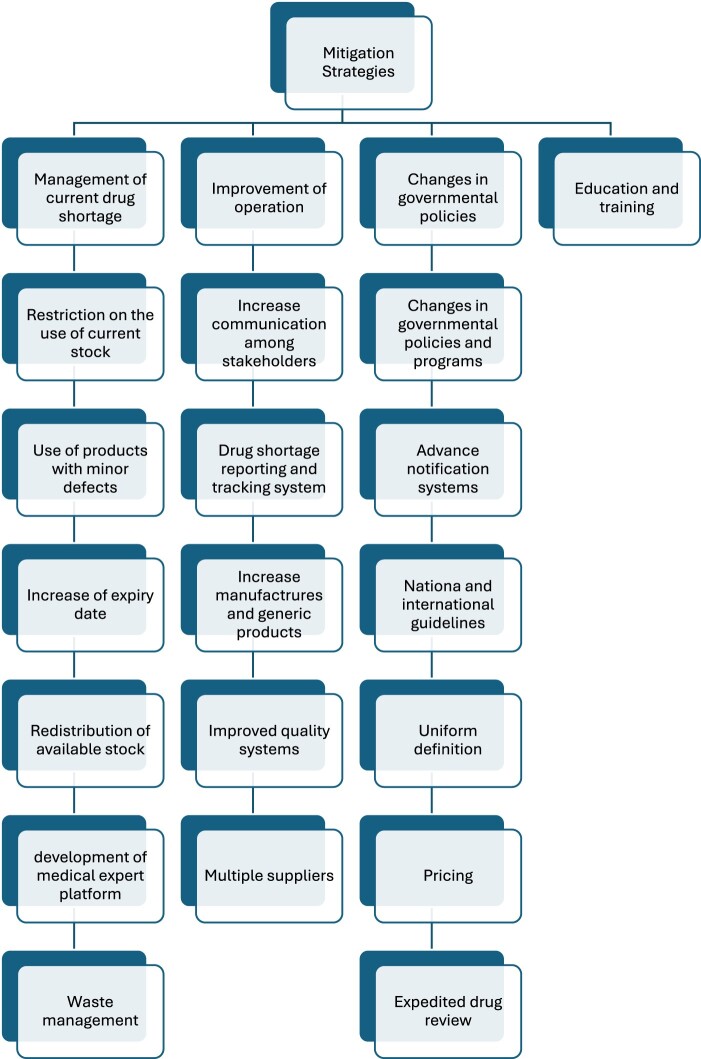
Mitigations measures suggested to tackle drug shortages in Lebanese hospitals.

In the current study, drug shortages forced hospitals to switch to more expensive alternative medicines to tackle this issue, resulting in a 60% increase in overall pharmaceutical costs at the hospitals, which also led to an 86.1% increase in costs at the patient level and a 91.7% increase at the pharmacy hospital level. Drug shortages usually result in an extra cost or budget for different stakeholders, especially patients, at all economic levels (Shukar et al., 2021). In high-income countries, stakeholders are aware of drug shortages, and suppliers have to manage the unavailability of raw materials through additional operations. Retailers are forced to purchase many drugs in short supply at increased prices or buy expensive alternative brands, start compounding or make logistic modifications. Hospitals have to put extra costs to manage the shortage, such as purchasing costly brands, excess inventories, and awareness programs to deliver staff knowledge. Studies estimated $200 million in purchasing expensive alternatives accompanied by additional labor costs of $359 million in United States hospitals due to drug shortage annually (Costelloe et al., 2015; Dill & Ahn, 2014; Food and Drug Administration, 2019; Fox & Tyler, 2013; Fox et al., 2014; Hughes et al., 2015; Mazer-Amirshahi et al., 2014; Phuong et al., 2019). On the other side, medicine prices tend to rise following a drug shortage, particularly for lower-priced generics, drugs with a single manufacturer, unapproved medicines, and orphan drugs (Dave et al., 2018; Fox & Tyler, 2017). An increase in medicine prices is also an illegal practice in the grey market, where large quantities of medications are stockpiled in advance and then sold at higher prices during shortages (Fittler et al., 2018; Jackson et al., 2012; Koenraadt & van de Ven, 2018), leading to a higher financial burden for patients. The out-of-the-pocket costs of patients increase as they have to purchase more expensive brands/alternatives/compounded medicines, pay more for a prolonged duration of therapy, extended hospital stay, and compromised treatment (Phuong et al., 2019). This was declared by oncology pharmacists in the United States in 2011 (Dill & Ahn, 2014). In low- and middle-income countries, few studies have been conducted and only reported increased expenses following drug shortages (Acosta et al., 2019; Bero et al., 2017; Fatima & Khaliq, 2017). Drug shortages are strongly influenced by the healthcare system, with high-income countries benefiting from models like Beveridge, Bismarck, and National Health Insurance, which reduce patient costs. In contrast, low- and middle-income countries rely on the Out-of-Pocket model, burdening patients financially (Kos, 2019). Figure 9 summarizes our recommendations to address drug shortages in Lebanese hospitals.

### Limitations

This study has its own limitations, including information bias since respondents relied on self-reported answers and questions might not be fully understood or estimated. A selection bias might be possible since not all hospitals in Lebanon are represented (although we did our best to include all major hospitals from all governorates in the country).

## Conclusion

In conclusion, drug shortages in Lebanon are driven by various factors and have been increasing across governorates. Overall, hospital pharmacists share similar attitudes toward the shortage, despite the fact that corrective actions might vary between them. The knowledge of drug shortages among hospital pharmacists is similar and most participants having access to the information needed about drug shortages.

The study highlights the urgent need for action, since medical departments have been suffering financially and mentally since the beginning of the drug shortage. Drug shortages in Lebanon are a national crisis and a threat to national security, with long-term consequences on medical management, patients’ lives and quality of care. In addition to imposing strict control over the pharmaceutical business to disable black markets, potential solutions to guarantee continuous drugs availability would be to incentivize and sponsor local pharmaceutical companies to produce accessible alternatives, as importation options have become limited. Meanwhile, physicians must adapt to these strange circumstances and treat with what is available until a solution arises.

## Supplementary Material

Appendix I.docx

## Declarations

### Availability of data and materials

All data generated or analyzed during this study are not publicly available. The dataset supporting the conclusions is available upon request to the corresponding author.
