# Supplementary material for: Perceptions, attitudes and experiences of hospital pharmacists working in the private sector regarding drug shortage in Lebanon: a national cross-sectional study
Source: J Pharm Policy Pract. 2025 Mar 10;18(1):2464786. doi: 10.1080/20523211.2025.2464786 (PMC11894753; doi:10.1080/20523211.2025.2464786)
Supplement: Appendix I.docx [file JPPP_A_2464786_SM9040.docx]

Table S1. Perceptions of the causes of drug shortages according to hospital pharmacists, overall and by gender.

|  |  | Overall (N=36) | Akkar (n=1) | Beirut (n=5) | Beqaa (n=3) | Mount Lebanon (n=12) | Nabatieh  (n=2) | North Lebanon (n=8) | South Lebanon (n=5) | p-value |
| --- | --- | --- | --- | --- | --- | --- | --- | --- | --- | --- |
| What do you think are the causes for drug shortages over the last 12 months? | | | | | | | | | | |
| Manufacturing problems | Often | 22 (61.1%) | 0 (0.0%) | 3 (60.0%) | 1 (33.3%) | 8 (66.7%) | 1 (50.0%) | 6 (75.0%) | 3 (60.0%) | 0.690 |
|  | Rarely | 2 (5.6%) | 0 (0.0%) | 0 (0.0%) | 0 (0.0%) | 2 (16.7%) | 0 (0.0%) | 0 (0.0%) | 0 (0.0%) |  |
|  | Sometimes | 12 (33.3%) | 1 (100.0%) | 2 (40.0%) | 2 (66.7%) | 2 (16.7%) | 1 (50.0%) | 2 (25.0%) | 2 (40.0%) |  |
|  |  |  |  |  |  |  |  |  |  |  |
| global shortage on active pharmaceutical ingredient (raw material) | Often | 21 (58.3%) | 0 (0.0%) | 3 (60.0%) | 1 (33.3%) | 8 (66.7%) | 1 (50.0%) | 5 (62.5%) | 3 (60.0%) | 0.761 |
|  | Rarely | 2 (5.6%) | 0 (0.0%) | 0 (0.0%) | 1 (33.3%) | 1 (8.3%) | 0 (0.0%) | 0 (0.0%) | 0 (0.0%) |  |
|  | Sometimes | 13 (36.1%) | 1 (100.0%) | 2 (40.0%) | 1 (33.3%) | 3 (25.0%) | 1 (50.0%) | 3 (37.5%) | 2 (40.0%) |  |
| Quality issues | Often | 3 (8.3%) | 0 (0.0%) | 1 (20.0%) | 0 (0.0%) | 1 (8.3%) | 0 (0.0%) | 1 (12.5%) | 0 (0.0%) | 0.913 |
|  | Sometimes | 33 (91.7%) | 1 (100.0%) | 4 (80.0%) | 3 (100.0%) | 11 (91.7%) | 2 (100.0%) | 7 (87.5%) | 5 (100.0%) |  |
| High quality recommendations from authorities | Often | 1 (2.8%) | 0 (0.0%) | 0 (0.0%) | 0 (0.0%) | 0 (0.0%) | 0 (0.0%) | 1 (12.5%) | 0 (0.0%) | 0.714 |
|  | Rarely | 4 (11.1%) | 0 (0.0%) | 0 (0.0%) | 0 (0.0%) | 2 (16.7%) | 1 (50.0%) | 1 (12.5%) | 0 (0.0%) |  |
|  | Sometimes | 31 (86.1%) | 1 (100.0%) | 5 (100.0%) | 3 (100.0%) | 10 (83.3%) | 1 (50.0%) | 6 (75.0%) | 5 (100.0%) |  |
| Poor prediction of the needs by the manufacturer | Often | 5 (13.9%) | 0 (0.0%) | 0 (0.0%) | 1 (33.3%) | 2 (16.7%) | 0 (0.0%) | 1 (12.5%) | 1 (20.0%) | 0.237 |
|  | Rarely | 2 (5.6%) | 0 (0.0%) | 2 (40.0%) | 0 (0.0%) | 0 (0.0%) | 0 (0.0%) | 0 (0.0%) | 0 (0.0%) |  |
|  | Sometimes | 29 (80.6%) | 1 (100.0%) | 3 (60.0%) | 2 (66.7%) | 10 (83.3%) | 2 (100.0%) | 7 (87.5%) | 4 (80.0%) |  |
| Economic crisis | Always | 36 (100.0%) | 1 (100.0%) | 5 (100.0%) | 3 (100.0%) | 12 (100.0%) | 2 (100.0%) | 8 (100.0%) | 5 (100.0%) | NA |
| Non-European production sites | Always | 28 (77.8%) | 0 (0.0%) | 2 (40.0%) | 2 (66.7%) | 10 (83.3%) | 2 (100.0%) | 7 (87.5%) | 5 (100.0%) | 0.418 |
|  | Often | 6 (16.7%) | 1 (100.0%) | 2 (40.0%) | 1 (33.3%) | 1 (8.3%) | 0 (0.0%) | 1 (12.5%) | 0 (0.0%) |  |
|  | Rarely | 1 (2.8%) | 0 (0.0%) | 1 (20.0%) | 0 (0.0%) | 0 (0.0%) | 0 (0.0%) | 0 (0.0%) | 0 (0.0%) |  |
|  | Sometimes | 1 (2.8%) | 0 (0.0%) | 0 (0.0%) | 0 (0.0%) | 1 (8.3%) | 0 (0.0%) | 0 (0.0%) | 0 (0.0%) |  |
| Parallel export/import | Always | 8 (22.2%) | 0 (0.0%) | 1 (20.0%) | 1 (33.3%) | 2 (16.7%) | 1 (50.0%) | 1 (12.5%) | 2 (40.0%) | 0.448 |
|  | Often | 11 (30.6%) | 0 (0.0%) | 3 (60.0%) | 0 (0.0%) | 6 (50.0%) | 0 (0.0%) | 1 (12.5%) | 1 (20.0%) |  |
|  | Rarely | 3 (8.3%) | 0 (0.0%) | 0 (0.0%) | 0 (0.0%) | 1 (8.3%) | 1 (50.0%) | 1 (12.5%) | 0 (0.0%) |  |
|  | Sometimes | 14 (38.9%) | 1 (100.0%) | 1 (20.0%) | 2 (66.7%) | 3 (25.0%) | 0 (0.0%) | 5 (62.5%) | 2 (40.0%) |  |
| Too many competitors | Always | 25 (69.4%) | 1 (100.0%) | 4 (80.0%) | 2 (66.7%) | 6 (50.0%) | 1 (50.0%) | 6 (75.0%) | 5 (100.0%) | 0.620 |
|  | Often | 7 (19.4%) | 0 (0.0%) | 0 (0.0%) | 1 (33.3%) | 3 (25.0%) | 1 (50.0%) | 2 (25.0%) | 0 (0.0%) |  |
|  | Sometimes | 4 (11.1%) | 0 (0.0%) | 1 (20.0%) | 0 (0.0%) | 3 (25.0%) | 0 (0.0%) | 0 (0.0%) | 0 (0.0%) |  |
| Too high prices | Always | 33 (91.7%) | 1 (100.0%) | 5 (100.0%) | 3 (100.0%) | 9 (75.0%) | 2 (100.0%) | 8 (100.0%) | 5 (100.0%) | 0.805 |
|  | Often | 1 (2.8%) | 0 (0.0%) | 0 (0.0%) | 0 (0.0%) | 1 (8.3%) | 0 (0.0%) | 0 (0.0%) | 0 (0.0%) |  |
|  | Sometimes | 2 (5.6%) | 0 (0.0%) | 0 (0.0%) | 0 (0.0%) | 2 (16.7%) | 0 (0.0%) | 0 (0.0%) | 0 (0.0%) |  |
| Too low prices | Always | 25 (69.4%) | 1 (100.0%) | 4 (80.0%) | 2 (66.7%) | 6 (50.0%) | 2 (100.0%) | 5 (62.5%) | 5 (100.0%) | 0.777 |
|  | Often | 5 (13.9%) | 0 (0.0%) | 1 (20.0%) | 0 (0.0%) | 3 (25.0%) | 0 (0.0%) | 1 (12.5%) | 0 (0.0%) |  |
|  | Sometimes | 6 (16.7%) | 0 (0.0%) | 0 (0.0%) | 1 (33.3%) | 3 (25.0%) | 0 (0.0%) | 2 (25.0%) | 0 (0.0%) |  |
| Tendering | Often | 7 (19.4%) | 0 (0.0%) | 0 (0.0%) | 0 (0.0%) | 3 (25.0%) | 1 (50.0%) | 1 (12.5%) | 2 (40.0%) | 0.553 |
|  | Rarely | 17 (47.2%) | 1 (100.0%) | 1 (20.0%) | 2 (66.7%) | 6 (50.0%) | 1 (50.0%) | 4 (50.0%) | 2 (40.0%) |  |
|  | Sometimes | 12 (33.3%) | 0 (0.0%) | 4 (80.0%) | 1 (33.3%) | 3 (25.0%) | 0 (0.0%) | 3 (37.5%) | 1 (20.0%) |  |
| Increasing demand for specific products | Often | 15 (41.7%) | 0 (0.0%) | 3 (60.0%) | 2 (66.7%) | 4 (33.3%) | 1 (50.0%) | 3 (37.5%) | 2 (40.0%) | 0.737 |
|  | Rarely | 6 (16.7%) | 0 (0.0%) | 1 (20.0%) | 1 (33.3%) | 1 (8.3%) | 1 (50.0%) | 1 (12.5%) | 1 (20.0%) |  |
|  | Sometimes | 15 (41.7%) | 1 (100.0%) | 1 (20.0%) | 0 (0.0%) | 7 (58.3%) | 0 (0.0%) | 4 (50.0%) | 2 (40.0%) |  |

Table S2. Attitudes of hospital pharmacists regarding drug shortages, overall and by governorates.

|  |  | Overall (N=36) | Akkar (n=1) | Beirut (n=5) | Beqaa (n=3) | Mount Lebanon (n=12) | Nabatieh (n=2) | North Lebanon (n=8) | South Lebanon (n=5) | p-value |
| --- | --- | --- | --- | --- | --- | --- | --- | --- | --- | --- |
| Impression of inequality in drug shortage | No | 29 (80.0%) | 1(100.0%) | 4 (80.0%) | 2 (66.7%) | 9 (75.0%) | 1 (50.0%) | 7 (87.5%) | 5(100.0%) | 0.943 |
|  | Yes | 7(19.5%) | 0 (0.0%) | 1 (20.0%) | 1 (33.3%) | 3 (25.0%) | 1 (50.0%) | 1 (12.5%) | 0 (0.0%) |  |
| Clinical impact of drug shortages | | | | | | | | | |  |
| Delay of therapy | Always | 16 (44.5%) | 1 (100.0%) | 2 (40.0%) | 1 (33.3%) | 4 (33.3%) | 2 (100.0%) | 5 (62.5%) | 1 (20.0%) | **0.032** |
|  | Often | 20 (55.6%) | 0 (0.0%) | 3 (60.0%) | 2 (66.7%) | 8 (66.7%) | 0 (0.0%) | 3 (37.5%) | 4 (80.0%) |  |
| Switch to lower dose | Often | 16 (44.4%) | 0 (0.0%) | 3 (60.0%) | 2 (66.7%) | 5 (41.7%) | 1 (50.0%) | 3 (37.5%) | 2 (40.0%) | 0.875 |
|  | Rarely | 4 (11.1%) | 0 (0.0%) | 1 (20.0%) | 0 (0.0%) | 3 (25.0%) | 0 (0.0%) | 0 (0.0%) | 0 (0.0%) |  |
|  | Sometimes | 16 (44.5%) | 1 (100.0%) | 1 (20.0%) | 1 (33.3%) | 4 (33.3%) | 1 (50.0%) | 5 (62.5%) | 3 (60.0%) |  |
| Substitution with alternative drug | Always | 12 (33.3%) | 1 (100.0%) | 2 (40.0%) | 1 (33.3%) | 0 (0.0%) | 2 (100.0%) | 5 (62.5%) | 1 (20.0%) | **0.039** |
|  | Often | 23 (63.9%) | 0 (0.0%) | 3 (60.0%) | 2 (66.7%) | 12 (100.0%) | 0 (0.0%) | 3 (37.5%) | 3 (60.0%) |  |
|  | Sometimes | 1 (2.8%) | 0 (0.0%) | 0 (0.0%) | 0 (0.0%) | 0 (0.0%) | 0 (0.0%) | 0 (0.0%) | 1 (20.0%) |  |
| Substitution with inferior drug | Often | 2 (5.6%) | 0 (0.0%) | 0 (0.0%) | 1 (33.3%) | 0 (0.0%) | 0 (0.0%) | 0 (0.0%) | 1 (20.0%) | 0.097 |
|  | Rarely | 4 (11.1%) | 1 (100.0%) | 1 (20.0%) | 0 (0.0%) | 2 (16.7%) | 0 (0.0%) | 0 (0.0%) | 0 (0.0%) |  |
|  | Sometimes | 30 (83.3%) | 0 (0.0%) | 4 (80.0%) | 2 (66.7%) | 10 (83.3%) | 2 (100.0%) | 8 (100.0%) | 4 (80.0%) |  |
| Medication errors | Often | 3 (8.3%) | 0 (0.0%) | 0 (0.0%) | 0 (0.0%) | 2 (16.7%) | 0 (0.0%) | 1 (12.5%) | 0 (0.0%) | 0.609 |
|  | Rarely | 8 (22.2%) | 1 (100.0%) | 2 (40.0%) | 1 (33.3%) | 3 (25.0%) | 0 (0.0%) | 0 (0.0%) | 1 (20.0%) |  |
|  | Sometimes | 25 (69.4%) | 0 (0.0%) | 3 (60.0%) | 2 (66.7%) | 7 (58.3%) | 2 (100.0%) | 7 (87.5%) | 4 (80.0%) |  |
| Referring patient to other hospital | Rarely | 26 (72.2%) | 1 (100.0%) | 5 (100.0%) | 1 (33.3%) | 8 (66.7%) | 2 (100.0%) | 7 (87.5%) | 2 (40.0%) | 0.171 |
|  | Sometimes | 10 (27.8%) | 0 (0.0%) | 0 (0.0%) | 2 (66.7%) | 4 (33.3%) | 0 (0.0%) | 1 (12.5%) | 3 (60.0%) |  |
| Rationing of the drug | Often | 1 (2.8%) | 0 (0.0%) | 0 (0.0%) | 0 (0.0%) | 1 (8.3%) | 0 (0.0%) | 0 (0.0%) | 0 (0.0%) | 0.899 |
|  | Rarely | 16 (44.4%) | 1 (100.0%) | 2 (40.0%) | 2 (66.7%) | 3 (25.0%) | 1 (50.0%) | 5 (62.5%) | 2 (40.0%) |  |
|  | Sometimes | 19 (52.8%) | 0 (0.0%) | 3 (60.0%) | 1 (33.3%) | 8 (66.7%) | 1 (50.0%) | 3 (37.5%) | 3 (60.0%) |  |
| Factors affected by food shortages | | | | | | | |  |  |  |
| Workload | Moderately affected | 10 (27.8%) | 1 (100.0%) | 1 (20.0%) | 1 (33.3%) | 5 (41.7%) | 0 (0.0%) | 1 (12.5%) | 1 (20.0%) | 0.446 |
|  | Severely affected | 26 (72.2%) | 0 (0.0%) | 4 (80.0%) | 2 (66.7%) | 7 (58.3%) | 2 (100.0%) | 7 (87.5%) | 4 (80.0%) |  |
| Stress of personnel | Little affected | 1 (2.8%) | 0 (0.0%) | 0 (0.0%) | 0 (0.0%) | 0 (0.0%) | 0 (0.0%) | 0 (0.0%) | 1 (20.0%) | 0.596 |
|  | Moderately affected | 29 (80.6%) | 1 (100.0%) | 5 (100.0%) | 2 (66.7%) | 9 (75.0%) | 2 (100.0%) | 6 (75.0%) | 4 (80.0%) |  |
|  | Severely affected | 6 (16.7%) | 0 (0.0%) | 0 (0.0%) | 1 (33.3%) | 3 (25.0%) | 0 (0.0%) | 2 (25.0%) | 0 (0.0%) |  |
| Relationship of pharmacy with physicians | little affected | 1 (2.8%) | 0 (0.0%) | 0 (0.0%) | 0 (0.0%) | 0 (0.0%) | 0 (0.0%) | 0 (0.0%) | 1 (20.0%) | 0.596 |
|  | Moderately affected | 29 (80.6%) | 1 (100.0%) | 5 (100.0%) | 2 (66.7%) | 9 (75.0%) | 2 (100.0%) | 6 (75.0%) | 4 (80.0%) |  |
|  | Severely affected | 6 (16.7%) | 0 (0.0%) | 0 (0.0%) | 1 (33.3%) | 3 (25.0%) | 0 (0.0%) | 2 (25.0%) | 0 (0.0%) |  |
| Relationship of pharmacy with nursing | Little affected | 27 (75.0%) | 1 (100.0%) | 5 (100.0%) | 3 (100.0%) | 8 (66.7%) | 2 (100.0%) | 5 (62.5%) | 3 (60.0%) | 0.496 |
|  | Moderately affected | 9 (25.0%) | 0 (0.0%) | 0 (0.0%) | 0 (0.0%) | 4 (33.3%) | 0 (0.0%) | 3 (37.5%) | 2 (40.0%) |  |
| Relationship of pharmacy with supplier | Little affected | 29 (80.6%) | 1 (100.0%) | 5 (100.0%) | 3 (100.0%) | 9 (75.0%) | 2 (100.0%) | 6 (75.0%) | 3 (60.0%) | 0.880 |
|  | Moderately affected | 6 (16.7%) | 0 (0.0%) | 0 (0.0%) | 0 (0.0%) | 2 (16.7%) | 0 (0.0%) | 2 (25.0%) | 2 (40.0%) |  |
|  | Severely affected | 1 (2.8%) | 0 (0.0%) | 0 (0.0%) | 0 (0.0%) | 1 (8.3%) | 0 (0.0%) | 0 (0.0%) | 0 (0.0%) |  |
| Relationship of hospital with patient | Little affected | 14 (38.9%) | 1 (100.0%) | 1 (20.0%) | 2 (66.7%) | 6 (50.0%) | 0 (0.0%) | 2 (25.0%) | 2 (40.0%) | 0.793 |
|  | Moderately affected | 20 (55.6%) | 0 (0.0%) | 4 (80.0%) | 1 (33.3%) | 5 (41.7%) | 2 (100.0%) | 5 (62.5%) | 3 (60.0%) |  |
|  | Severely affected | 2 (5.6%) | 0 (0.0%) | 0 (0.0%) | 0 (0.0%) | 1 (8.3%) | 0 (0.0%) | 1 (12.5%) | 0 (0.0%) |  |

Table S3. Practices related to drug shortages as reported by hospital pharmacists, overall and by governorates.

|  |  | Overall (N=36) | Akkar  (n=1) | Beirut  (n=5) | Beqaa (n=3) | Mount Lebanon (n=12) | Nabatieh (n=2) | North Lebanon (n=8) | South Lebanon (n=5) | p-value |  |
| --- | --- | --- | --- | --- | --- | --- | --- | --- | --- | --- | --- |
| tendering purchasing | Yes | 36 (100.0%) | 1 (100.0%) | 5 (100.0%) | 3 (100.0%) | 12 (100.0%) | 2 (100.0%) | 8 (100.0%) | 5 (100.0%) | NA |  |
|  |  |  |  |  |  |  |  |  |  |  |  |
| In case of drug shortage, is there usually an alternative treatment or product available? | Yes, often | 24 (66.7%) | 1 (100.0%) | 4 (80.0%) | 3 (100.0%) | 9 (75.0%) | 1 (50.0%) | 2 (25.0%) | 4 (80.0%) | 0.139 |  |
|  | Yes, sometime | 12 (33.3%) | 0 (0.0%) | 1 (20.0%) | 0 (0.0%) | 3 (25.0%) | 1 (50.0%) | 6 (75.0%) | 1 (20.0%) |  |  |
| In case of a drug shortage, is an effort made by the manufacturer | Yes, always | 1 (2.8%) | 0 (0.0%) | 1 (20.0%) | 0 (0.0%) | 0 (0.0%) | 0 (0.0%) | 0 (0.0%) | 0 (0.0%) | 0.451 |  |
|  | Yes, often | 12 (33.3%) | 1 (100.0%) | 1 (20.0%) | 1 (33.3%) | 5 (41.7%) | 0 (0.0%) | 1 (12.5%) | 3 (60.0%) |  |  |
|  | Yes, sometime | 21 (58.3%) | 0 (0.0%) | 3 (60.0%) | 2 (66.7%) | 5 (41.7%) | 2 (100.0%) | 7 (87.5%) | 2 (40.0%) |  |  |
|  | Yes, sometimes | 2 (5.6%) | 0 (0.0%) | 0 (0.0%) | 0 (0.0%) | 2 (16.7%) | 0 (0.0%) | 0 (0.0%) | 0 (0.0%) |  |  |
| Class of drugs affected | | | | | | |  |  |  |  |  |
| Gastro-intestinal diseases | Yes | 30 (83.3%) | 1 (100.0%) | 4 (80.0%) | 2 (66.7%) | 9 (75.0%) | 2 (100.0%) | 7 (87.5%) | 5 (100.0%) | 0.816 |  |
| Cardiovascular diseases | Yes | 8 (22.2%) | 0 (0.0%) | 1 (20.0%) | 0 (0.0%) | 4 (33.3%) | 0 (0.0%) | 0 (0.0%) | 3 (60.0%) | 0.174 |  |
| Central nervous system | Yes | 9 (25.0%) | 0 (0.0%) | 0 (0.0%) | 0 (0.0%) | 6 (50.0%) | 0 (0.0%) | 1 (12.5%) | 2 (40.0%) | 0.177 |  |
| Anti-infective | Yes | 9 (25.0%) | 0 (0.0%) | 1 (20.0%) | 0 (0.0%) | 5 (41.7%) | 1 (50.0%) | 2 (25.0%) | 0 (0.0%) | 0.48 |  |
| Endocrine system | Yes | 7 (19.4%) | 0 (0.0%) | 2 (40.0%) | 0 (0.0%) | 1 (8.3%) | 0 (0.0%) | 2 (25.0%) | 2 (40.0%) | 0.512 |  |
| Anesthesia | Yes | 36 (100.0%) | 1 (100.0%) | 5 (100.0%) | 3 (100.0%) | 12 (100.0%) | 2 (100.0%) | 8 (100.0%) | 5 (100.0%) | NA |  |
| Skin diseases | Yes | 23 (63.9%) | 0 (0.0%) | 4 (80.0%) | 2 (66.7%) | 8 (66.7%) | 1 (50.0%) | 4 (50.0%) | 4 (80.0%) | 0.706 |  |
| Obstetrics, gynecology and urinary tract disorders | NR | 36 (100.0%) | 1 (100.0%) | 5 (100.0%) | 3 (100.0%) | 12 (100.0%) | 2 (100.0%) | 8 (100.0%) | 5 (100.0%) | NA |  |
| Anti-cancer | NR | 36 (100.0%) | 1 (100.0%) | 5 (100.0%) | 3 (100.0%) | 12 (100.0%) | 2 (100.0%) | 8 (100.0%) | 5 (100.0%) | NA |  |
| Nutrition and blood diseases | NR | 36 (100.0%) | 1 (100.0%) | 5 (100.0%) | 3 (100.0%) | 12 (100.0%) | 2 (100.0%) | 8 (100.0%) | 5 (100.0%) | NA |  |
| Musculoskeletal and joint diseases | NR | 36 (100.0%) | 1 (100.0%) | 5 (100.0%) | 3 (100.0%) | 12 (100.0%) | 2 (100.0%) | 8 (100.0%) | 5 (100.0%) | NA |  |
| Eye disorders | NR | 36 (100.0%) | 1 (100.0%) | 5 (100.0%) | 3 (100.0%) | 12 (100.0%) | 2 (100.0%) | 8 (100.0%) | 5 (100.0%) | NA |  |
| Ear, nose and oropharynx disorders | NR | 36 (100.0%) | 1 (100.0%) | 5 (100.0%) | 3 (100.0%) | 12 (100.0%) | 2 (100.0%) | 8 (100.0%) | 5 (100.0%) | NA |  |
| Immunological products and vaccines | NR | 36 (100.0%) | 1 (100.0%) | 5 (100.0%) | 3 (100.0%) | 12 (100.0%) | 2 (100.0%) | 8 (100.0%) | 5 (100.0%) | NA |  |
| Respiratory system | NR | 36 (100.0%) | 1 (100.0%) | 5 (100.0%) | 3 (100.0%) | 12 (100.0%) | 2 (100.0%) | 8 (100.0%) | 5 (100.0%) | NA |  |
| Are the affected drugs mostly generic or originator? | | | | | | | | | | | |
| Gastro-intestinal diseases | Generic | 30 (83.3%) | 1 (100.0%) | 4 (80.0%) | 2 (66.7%) | 9 (75.0%) | 2 (100.0%) | 7 (87.5%) | 5 (100.0%) | 0.816 |  |
| Cardiovascular diseases | Generic | 8 (22.2%) | 0 (0.0%) | 1 (20.0%) | 0 (0.0%) | 4 (33.3%) | 0 (0.0%) | 0 (0.0%) | 3 (60.0%) | 0.174 |  |
| Respiratory system | NR | 36 (100.0%) | 1 (100.0%) | 5 (100.0%) | 3 (100.0%) | 12 (100.0%) | 2 (100.0%) | 8 (100.0%) | 5 (100.0%) | NA |  |
| Central nervous system | Originator/generic | 10 (27.8%) | 0 (0.0%) | 0 (0.0%) | 0 (0.0%) | 6 (50.0%) | 1 (50.0%) | 1 (12.5%) | 2 (40.0%) | 0.223 |  |
| Anti-infective | Originator/generic | 8 (22.2%) | 0 (0.0%) | 1 (20.0%) | 0 (0.0%) | 5 (41.7%) | 0 (0.0%) | 2 (25.0%) | 0 (0.0%) | 0.444 |  |
| Endocrine system | Originator | 7 (19.4%) | 0 (0.0%) | 2 (40.0%) | 0 (0.0%) | 1 (8.3%) | 0 (0.0%) | 2 (25.0%) | 2 (40.0%) | 0.512 |  |
| Skin diseases | Generic | 23 (63.9%) | 0 (0.0%) | 4 (80.0%) | 2 (66.7%) | 8 (66.7%) | 1 (50.0%) | 4 (50.0%) | 4 (80.0%) | 0.706 |  |
| Anesthesia | Originator/generic | 36 (100.0%) | 1 (100.0%) | 5 (100.0%) | 3 (100.0%) | 12 (100.0%) | 2 (100.0%) | 8 (100.0%) | 5 (100.0%) | NA |  |
| Obstetrics, gynecology and urinary tract disorders | NR | 36 (100.0%) | 1 (100.0%) | 5 (100.0%) | 3 (100.0%) | 12 (100.0%) | 2 (100.0%) | 8 (100.0%) | 5 (100.0%) | NA |  |
| Anti-cancer | NR | 36 (100.0%) | 1 (100.0%) | 5 (100.0%) | 3 (100.0%) | 12 (100.0%) | 2 (100.0%) | 8 (100.0%) | 5 (100.0%) | NA |  |
| Nutrition and blood diseases | NR | 36 (100.0%) | 1 (100.0%) | 5 (100.0%) | 3 (100.0%) | 12 (100.0%) | 2 (100.0%) | 8 (100.0%) | 5 (100.0%) | NA |  |
| Musculoskeletal and joint diseases | NR | 36 (100.0%) | 1 (100.0%) | 5 (100.0%) | 3 (100.0%) | 12 (100.0%) | 2 (100.0%) | 8 (100.0%) | 5 (100.0%) | NA |  |
| Eye disorders | NR | 36 (100.0%) | 1 (100.0%) | 5 (100.0%) | 3 (100.0%) | 12 (100.0%) | 2 (100.0%) | 8 (100.0%) | 5 (100.0%) | NA |  |
| Ear, nose and oropharynx disorders | NR | 36 (100.0%) | 1 (100.0%) | 5 (100.0%) | 3 (100.0%) | 12 (100.0%) | 2 (100.0%) | 8 (100.0%) | 5 (100.0%) | NA |  |
| Immunological products and vaccines | NR | 36 (100.0%) | 1 (100.0%) | 5 (100.0%) | 3 (100.0%) | 12 (100.0%) | 2 (100.0%) | 8 (100.0%) | 5 (100.0%) | NA |  |

Cont’ Table S3. Practices related to drug shortages as reported by hospital pharmacists, overall and by governorates.

|  |  | Overall (N=36) | Akkar  (n=1) | Beirut  (n=5) | Beqaa (n=3) | | Mount Lebanon (n=12) | | Nabatieh (n=2) | | North Lebanon (n=8) | | South Lebanon (n=5) | | p-value | |  |
| --- | --- | --- | --- | --- | --- | --- | --- | --- | --- | --- | --- | --- | --- | --- | --- | --- | --- |
| Are the affected drugs mostly cheap or expensive? | | | | | | | | | | | | | | | | |  |
| Gastro-intestinal diseases | Cheap | 30 (83.3%) | 1 (100.0%) | 4 (80.0%) | 2 (66.7%) | | 9 (75.0%) | | 2 (100.0%) | | 7 (87.5%) | | 5 (100.0%) | | 0.816 | |  |
| Cardiovascular diseases | Cheap/expensive | 8 (22.2%) | 0 (0.0%) | 1 (20.0%) | 0 (0.0%) | | 4 (33.3%) | | 0 (0.0%) | | 0 (0.0%) | | 3 (60.0%) | | 0.174 | |  |
| Central nervous system | Cheap/expensive | 10 (27.8%) | 0 (0.0%) | 0 (0.0%) | 0 (0.0%) | | 6 (50.0%) | | 1 (50.0%) | | 1 (12.5%) | | 2 (40.0%) | | 0.223 | |  |
| Anti-infective | Cheap | 8 (22.2%) | 0 (0.0%) | 1 (20.0%) | 0 (0.0%) | | 5 (41.7%) | | 0 (0.0%) | | 2 (25.0%) | | 0 (0.0%) | | 0.444 | |  |
| Skin diseases | Expensive | 24 (66.7%) | 0 (0.0%) | 4 (80.0%) | 2 (66.7%) | | 8 (66.7%) | | 1 (50.0%) | | 5 (62.5%) | | 4 (80.0%) | | 0.795 | |  |
| Endocrine system | Cheap/expensive | 7 (19.4%) | 0 (0.0%) | 2 (40.0%) | 0 (0.0%) | | 1 (8.3%) | | 0 (0.0%) | | 2 (25.0%) | | 2 (40.0%) | | 0.512 | |  |
| Anesthesia | Cheap | 36 (100.0%) | 1 (100.0%) | 5 (100.0%) | 3 (100.0%) | | 12 (100.0%) | | 2 (100.0%) | | 8 (100.0%) | | 5 (100.0%) | | NA | |  |
| Respiratory system | NR | 36 (100.0%) | 1 (100.0%) | 5 (100.0%) | 3 (100.0%) | | 12 (100.0%) | | 2 (100.0%) | | 8 (100.0%) | | 5 (100.0%) | | NA | |  |
| Obstetrics, gynecology and urinary tract disorders | NR | 36 (100.0%) | 1 (100.0%) | 5 (100.0%) | 3 (100.0%) | | 12 (100.0%) | | 2 (100.0%) | | 8 (100.0%) | | 5 (100.0%) | | NA | |  |
| Anti-cancer | NR | 36 (100.0%) | 1 (100.0%) | 5 (100.0%) | 3 (100.0%) | | 12 (100.0%) | | 2 (100.0%) | | 8 (100.0%) | | 5 (100.0%) | | NA | |  |
| Nutrition and blood diseases | NR | 36 (100.0%) | 1 (100.0%) | 5 (100.0%) | 3 (100.0%) | | 12 (100.0%) | | 2 (100.0%) | | 8 (100.0%) | | 5 (100.0%) | | NA | |  |
| Musculoskeletal and joint diseases | NR | 36 (100.0%) | 1 (100.0%) | 5 (100.0%) | 3 (100.0%) | | 12 (100.0%) | | 2 (100.0%) | | 8 (100.0%) | | 5 (100.0%) | | NA | |  |
| Eye disorders | NR | 36 (100.0%) | 1 (100.0%) | 5 (100.0%) | 3 (100.0%) | | 12 (100.0%) | | 2 (100.0%) | | 8 (100.0%) | | 5 (100.0%) | | NA | |  |
| Ear, nose and oropharynx disorders | NR | 36 (100.0%) | 1 (100.0%) | 5 (100.0%) | 3 (100.0%) | | 12 (100.0%) | | 2 (100.0%) | | 8 (100.0%) | | 5 (100.0%) | | NA | |  |
| Immunological products and vaccines | NR | 36 (100.0%) | 1 (100.0%) | 5 (100.0%) | 3 (100.0%) | | 12 (100.0%) | | 2 (100.0%) | | 8 (100.0%) | | 5 (100.0%) | | NA | |  |
|  |  |  |  |  |  | |  | |  | |  | |  | |  | |  |
| What is the most affected form? | | | | | |  | |  | |  | |  | |  | |  | |
| Gastro-intestinal diseases | Oral | 30 (83.3%) | 1 (100.0%) | 4 (80.0%) | 2 (66.7%) | | 9 (75.0%) | | 2 (100.0%) | | 7 (87.5%) | | 5 (100.0%) | | 0.816 | |  |
| Cardiovascular diseases | Oral | 8 (22.2%) | 0 (0.0%) | 1 (20.0%) | 0 (0.0%) | | 4 (33.3%) | | 0 (0.0%) | | 0 (0.0%) | | 3 (60.0%) | | 0.174 | |  |
| Anesthesia | Oral/injectable | 36 (100.0%) | 1 (100.0%) | 5 (100.0%) | 3 (100.0%) | | 12 (100.0%) | | 2 (100.0%) | | 8 (100.0%) | | 5 (100.0%) | | NA | |  |
| Central nervous system | Oral/injectable | 10 (27.8%) | 0 (0.0%) | 0 (0.0%) | 0 (0.0%) | | 6 (50.0%) | | 1 (50.0%) | | 1 (12.5%) | | 2 (40.0%) | | 0.223 | |  |
| Anti-infective | Oral | 2 (5.6%) | 0 (0.0%) | 0 (0.0%) | 0 (0.0%) | | 2 (16.7%) | | 0 (0.0%) | | 0 (0.0%) | | 0 (0.0%) | | 0.786 | |  |
|  | Oral/dermal | 6 (16.7%) | 0 (0.0%) | 1 (20.0%) | 0 (0.0%) | | 3 (25.0%) | | 0 (0.0%) | | 2 (25.0%) | | 0 (0.0%) | |  |  |  |
| Endocrine system | Oral | 7 (19.4%) | 0 (0.0%) | 2 (40.0%) | 0 (0.0%) | | 1 (8.3%) | | 0 (0.0%) | | 2 (25.0%) | | 2 (40.0%) | | 0.512 | |  |
| Skin diseases | Dermal | 23 (63.9%) | 0 (0.0%) | 4 (80.0%) | 2 (66.7%) | | 8 (66.7%) | | 1 (50.0%) | | 4 (50.0%) | | 4 (80.0%) | | 0.706 | |  |
| Respiratory system | NR | 36 (100.0%) | 1 (100.0%) | 5 (100.0%) | 3 (100.0%) | | 12 (100.0%) | | 2 (100.0%) | | 8 (100.0%) | | 5 (100.0%) | | NA | |  |
| Obstetrics,gynecology and urinary tract disorders | NR | 36 (100.0%) | 1 (100.0%) | 5 (100.0%) | 3 (100.0%) | | 12 (100.0%) | | 2 (100.0%) | | 8 (100.0%) | | 5 (100.0%) | | NA | |  |
| Anti-cancer | NR | 36 (100.0%) | 1 (100.0%) | 5 (100.0%) | 3 (100.0%) | | 12 (100.0%) | | 2 (100.0%) | | 8 (100.0%) | | 5 (100.0%) | | NA | |  |
| Nutrition and blood diseases | NR | 36 (100.0%) | 1 (100.0%) | 5 (100.0%) | 3 (100.0%) | | 12 (100.0%) | | 2 (100.0%) | | 8 (100.0%) | | 5 (100.0%) | | NA | |  |
| Musculoskeletal and joint diseases | NR | 36 (100.0%) | 1 (100.0%) | 5 (100.0%) | 3 (100.0%) | | 12 (100.0%) | | 2 (100.0%) | | 8 (100.0%) | | 5 (100.0%) | | NA | |  |
| Eye disorders | NR | 36 (100.0%) | 1 (100.0%) | 5 (100.0%) | 3 (100.0%) | | 12 (100.0%) | | 2 (100.0%) | | 8 (100.0%) | | 5 (100.0%) | | NA | |  |
| Ear, nose and oropharynx disorders | NR | 36 (100.0%) | 1 (100.0%) | 5 (100.0%) | 3 (100.0%) | | 12 (100.0%) | | 2 (100.0%) | | 8 (100.0%) | | 5 (100.0%) | | NA | |  |
| Immunological products and vaccines | NR | 36 (100.0%) | 1 (100.0%) | 5 (100.0%) | 3 (100.0%) | | 12 (100.0%) | | 2 (100.0%) | | 8 (100.0%) | | 5 (100.0%) | | NA | |  |

Table S4. Source of information related to drug shortages in Lebanese hospital pharmacies, overall and by governorates.

|  |  | Overall (N=36) | Akkar  (n=1) | Beirut  (n=5) | Beqaa (n=3) | Mount Lebanon (n=12) | Nabatieh (n=2) | North Lebanon (n=8) | South Lebanon (n=5) | p-value |
| --- | --- | --- | --- | --- | --- | --- | --- | --- | --- | --- |
| Sources of information |  |  |  |  |  |  |  |  |  |  |
| Government | Never | 12 (33.3%) | 0 (0.0%) | 4 (80.0%) | 0 (0.0%) | 1 (8.3%) | 0 (0.0%) | 5 (62.5%) | 2 (40.0%) | 0.007 |
|  | Rarely | 22 (61.1%) | 1 (100.0%) | 1 (20.0%) | 3 (100.0%) | 11 (91.7%) | 1 (50.0%) | 2 (25.0%) | 3 (60.0%) |  |
|  | always | 1 (2.8%) | 0 (0.0%) | 0 (0.0%) | 0 (0.0%) | 0 (0.0%) | 1 (50.0%) | 0 (0.0%) | 0 (0.0%) |  |
|  | Sometimes | 1 (2.8%) | 0 (0.0%) | 0 (0.0%) | 0 (0.0%) | 0 (0.0%) | 0 (0.0%) | 1 (12.5%) | 0 (0.0%) |  |
| Wholesaler | Always | 29 (80.6%) | 1 (100.0%) | 5 (100.0%) | 2 (66.7%) | 7 (58.3%) | 2 (100.0%) | 8 (100.0%) | 4 (80.0%) | 0.237 |
|  | Often | 7 (19.4%) | 0 (0.0%) | 0 (0.0%) | 1 (33.3%) | 5 (41.7%) | 0 (0.0%) | 0 (0.0%) | 1 (20.0%) |  |
| Pharmaceutical company | Always | 26 (72.2%) | 1 (100.0%) | 4 (80.0%) | 2 (66.7%) | 7 (58.3%) | 1 (50.0%) | 7 (87.5%) | 4 (80.0%) | 0.769 |
|  | Often | 10 (27.8%) | 0 (0.0%) | 1 (20.0%) | 1 (33.3%) | 5 (41.7%) | 1 (50.0%) | 1 (12.5%) | 1 (20.0%) |  |
| Other hospital | Always | 21 (58.3%) | 1 (100.0%) | 4 (80.0%) | 1 (33.3%) | 5 (41.7%) | 1 (50.0%) | 6 (75.0%) | 3 (60.0%) | 0.696 |
|  | Often | 8 (22.2%) | 0 (0.0%) | 1 (20.0%) | 1 (33.3%) | 4 (33.3%) | 0 (0.0%) | 1 (12.5%) | 1 (20.0%) |  |
|  | Rarely | 2 (5.6%) | 0 (0.0%) | 0 (0.0%) | 0 (0.0%) | 1 (8.3%) | 1 (50.0%) | 0 (0.0%) | 0 (0.0%) |  |
|  | Sometimes | 5 (13.9%) | 0 (0.0%) | 0 (0.0%) | 1 (33.3%) | 2 (16.7%) | 0 (0.0%) | 1 (12.5%) | 1 (20.0%) |  |
| Professional association | Always | 1 (2.8%) | 0 (0.0%) | 0 (0.0%) | 0 (0.0%) | 1 (8.3%) | 0 (0.0%) | 0 (0.0%) | 0 (0.0%) | 0.927 |
|  | Never | 17 (47.2%) | 1 (100.0%) | 2 (40.0%) | 2 (66.7%) | 6 (50.0%) | 1 (50.0%) | 2 (25.0%) | 3 (60.0%) |  |
|  | Often | 5 (13.9%) | 0 (0.0%) | 1 (20.0%) | 1 (33.3%) | 0 (0.0%) | 0 (0.0%) | 3 (37.5%) | 0 (0.0%) |  |
|  | Rarely | 9 (25.0%) | 0 (0.0%) | 2 (40.0%) | 0 (0.0%) | 3 (25.0%) | 1 (50.0%) | 2 (25.0%) | 1 (20.0%) |  |
|  | Sometimes | 4 (11.1%) | 0 (0.0%) | 0 (0.0%) | 0 (0.0%) | 2 (16.7%) | 0 (0.0%) | 1 (12.5%) | 1 (20.0%) |  |
| No information was given | Never | 22 (61.1%) | 1 (100.0%) | 2 (40.0%) | 2 (66.7%) | 9 (75.0%) | 1 (50.0%) | 4 (50.0%) | 3 (60.0%) | 0.411 |
|  | Rarely | 8 (22.2%) | 0 (0.0%) | 2 (40.0%) | 0 (0.0%) | 1 (8.3%) | 1 (50.0%) | 4 (50.0%) | 0 (0.0%) |  |
|  | Sometimes | 6 (16.7%) | 0 (0.0%) | 1 (20.0%) | 1 (33.3%) | 2 (16.7%) | 0 (0.0%) | 0 (0.0%) | 2 (40.0%) |  |
| Channels approached during drug shortages |  |  |  |  |  |  |  |  |  |  |
| Pharmaceutical company | Always | 19 (52.8%) | 0 (0.0%) | 2 (40.0%) | 2 (66.7%) | 8 (66.7%) | 2 (100.0%) | 3 (37.5%) | 2 (40.0%) | 0.48 |
|  | Often | 17 (47.2%) | 1 (100.0%) | 3 (60.0%) | 1 (33.3%) | 4 (33.3%) | 0 (0.0%) | 5 (62.5%) | 3 (60.0%) |  |
| Other hospital | Always | 10 (27.8%) | 0 (0.0%) | 1 (20.0%) | 0 (0.0%) | 5 (41.7%) | 1 (50.0%) | 2 (25.0%) | 1 (20.0%) | 0.70 |
|  | Often | 19 (52.8%) | 1 (100.0%) | 3 (60.0%) | 1 (33.3%) | 5 (41.7%) | 1 (50.0%) | 5 (62.5%) | 3 (60.0%) |  |
|  | Rarely | 2 (5.6%) | 0 (0.0%) | 1 (20.0%) | 0 (0.0%) | 1 (8.3%) | 0 (0.0%) | 0 (0.0%) | 0 (0.0%) |  |
|  | Sometimes | 5 (13.9%) | 0 (0.0%) | 0 (0.0%) | 2 (66.7%) | 1 (8.3%) | 0 (0.0%) | 1 (12.5%) | 1 (20.0%) |  |
| Another wholesaler | Always | 18 (52.8%) | 0 (0.0%) | 2 (40.0%) | 2 (66.6%) | 6 (50.0%) | 2 (100.0%) | 5 (62.5%) | 2 (40.0%) | 0.22 |
|  | Often | 17 (47.2%) | 1 (100.0%) | 3 (60.0%) | 1 (33.3%) | 6 (50.0%) | 0 (0.0%) | 3 (37.5%) | 3 (60.0%) |  |
| Parallel import | Always | 5 (13.9%) | 0 (0.0%) | 0 (0.0%) | 0 (0.0%) | 2 (16.7%) | 1 (50.0%) | 0 (0.0%) | 2 (40.0%) | 0.61 |
|  | Often | 27 (75.0%) | 1 (100.0%) | 5 (100.0%) | 2 (66.7%) | 7 (58.3%) | 1 (50.0%) | 8 (100.0%) | 3 (60.0%) |  |
|  | Rarely | 1 (2.8%) | 0 (0.0%) | 0 (0.0%) | 0 (0.0%) | 1 (8.3%) | 0 (0.0%) | 0 (0.0%) | 0 (0.0%) |  |
|  | Sometimes | 3 (8.3%) | 0 (0.0%) | 0 (0.0%) | 1 (33.3%) | 2 (16.7%) | 0 (0.0%) | 0 (0.0%) | 0 (0.0%) |  |
